# Role of reactive oxygen species in brucein D-mediated p38-mitogen-activated protein kinase and nuclear factor-*κ*B signalling pathways in human pancreatic adenocarcinoma cells

**DOI:** 10.1038/sj.bjc.6605487

**Published:** 2010-01-12

**Authors:** S T Lau, Z X Lin, P S Leung

**Affiliations:** 1 School of Biomedical Sciences, Faculty of Medicine, The Chinese University of Hong Kong, Shatin, New Territories, Hong Kong, China; 2 School of Chinese Medicine, Faculty of Science, The Chinese University of Hong Kong, Shatin, New Territories, Hong Kong, China

**Keywords:** brucein D, PANC-1 cells, apoptosis, reactive oxygen species, pancreatic cancer

## Abstract

**Background:**

In human pancreatic adenocarcinoma, nuclear factor-kappa-B (NF-*κ*B) transcription factor is constitutively activated that contributes to the resistance of the tumour cells to induced apoptosis. In our earlier studies, we have shown that brucein D (BD) mediated apoptosis through activation of the p38-mitogen-activated protein kinase (MAPK) signalling pathway in pancreatic cancer cells. This study investigated the function of reactive oxygen species (ROS) in BD-mediated p38-MAPK and NF-*κ*B signalling pathways in PANC-1 cells.

**Methods:**

Glutathione and dihydroethidium assays were used to measure the antioxidant and superoxide levels, respectively. The protein expression of p22^phox^, p67^phox^ and p38-MAPK were examined by western blot. The NF-*κ*B activity was evaluated by electrophoretic mobility shift assay.

**Results:**

Treatment with BD depleted the intracellular glutathione levels in PANC-1 cells. Brucein D triggered the activation of NADPH oxidase isoforms, p22^phox^ and p67^phox^ while enhancing the generation of superoxide. Increases in both intracellular ROS and NADPH oxidase activity were inhibited by an antioxidant, *N*-acetylcysteine (NAC). Brucein D-mediated activation of p38-MAPK was also inhibited by NAC. However, inhibition of NF-*κ*B activity in BD-treated cells was independent of ROS. *In vivo* studies showed that BD treatment effectively reduced the rate of xenograft human pancreatic tumour in nude mice with no significant toxicity.

**Conclusion:**

These data suggest that BD is an apoptogenic agent for pancreatic cancer cells through activation of the redox-sensitive p38-MAPK pathway and inhibition of NF-*κ*B anti-apoptotic activity in pancreatic cancer cells.

Human pancreatic adenocarcinoma is a highly aggressive malignancy intrinsically resistant to radiotherapy and chemotherapy (Wanebo and Vezeridis, 1996). It is well known that radio- and chemotherapy are to destroy cancer cells through an induction of cellular oxidative stress (Lau *et al*, 2008a). Oxidative stress is the result of enhanced production of intracellular reactive oxygen species (ROS) and/or impaired function of the cellular antioxidant defence system (Buttke and Sandstrom, 1994). Reactive oxygen species are highly reactive O_2_ metabolites, including superoxide radicals (O_2_^•−^), hydrogen peroxide (H_2_O_2_) and hydroxyl radicals (OH^•^) (Thannickal and Fanburg, 2000); they serve as key secondary messengers in numerous signalling pathways, eliciting a diverse array of biological responses such as transcriptional regulation, differentiation, proliferation and oncogenic transformation (Curtin *et al*, 2002; Martindale and Holbrook, 2002; Storz, 2006). Cellular ROS are thus essential to cell survival. On the other hand, ROS also have an important function in pro-apoptotic pathways (Curtin *et al*, 2002; Matsuzawa and Ichijo, 2005). As intracellular redox status is critical in determining the survival and death of cells (Hampton and Orrenius, 1998), many cancer therapeutics target to induce cellular apoptosis by disrupting the redox balance and depleting the intracellular thiol buffer system through extrusion or redistribution of glutathione (GSH) (Ghibelli *et al*, 1995).

One of the contributing factors to the aggressiveness, unresponsiveness and high mortality rate of pancreatic adenocarcinoma is the resistance of malignant pancreatic cells to apoptosis induced by radio- and chemo-therapeutic therapy (Wanebo and Vezeridis, 1996; Liptay *et al*, 2003). It has been well established that the transcription factor nuclear factor-kappa-B (NF-*κ*B) mediates a central signalling pathway resulting in protection from apoptotic cell death (Karin and Lin, 2002; Kucharczak *et al*, 2003). There is mounting evidence to suggest that constitutive activation of anti-apoptotic proteins, such as NF-*κ*B (Aggarwal, 2000), signal transducers and activators of transcription proteins (Battle and Frank, 2002), heat shock proteins (Beere and Green, 2001) and phosphatidylinositide-3-kinase (Nicholson and Anderson, 2002) all have a function in the resistance of pancreatic cancer cells to apoptosis. Nuclear factor-*κ*B is a downstream effector of several growth receptors and is constitutively activated in human pancreatic tumour cells (Wang *et al*, 1999). Nuclear factor-*κ*B is normally sequestered in the cytosol in its inactive form by inhibitory I*κ*B proteins. Inducible phosphorylation of I*κ*Bs by IKKs targets for polyubiquitination and subsequent degradation, leading to activation of NF-*κ*B (Yip-Schneider *et al*, 2005). The active NF-*κ*B complex is a homo- or heterodimer composed of proteins from the NF-*κ*B/Rel family, including NF-*κ*B_1_ (p50/105), NF-*κ*B_2_ (p52/100), RelA (p65), RelB and c-Rel (Verma *et al*, 1995; Baldwin, 1996). Complex assembly of c-Rel, RelA and RelB with their inhibitors, the I*κ*Bs, in the cytoplasm results in inactivity as the interaction masks the nuclear localisation signal of the NF-*κ*B family members (Sen and Baltimore, 1986; Baeuerle and Baltimore, 1988). Several I*κ*B proteins have been identified, such as I*κ*B-*α*, I*κ*B-*β* and I*κ*B-*ε* (Verma *et al*, 1995; Whiteside and Israel, 1997). On stimulation, I*κ*B is phosphorylated by I*κ*B kinase, leading to its rapid degradation. Consequently, NF-*κ*B proteins are released and translocate into the nucleus in which they transactivate target genes (Baldwin, 1996; Gilmore *et al*, 1996; Ghosh and Karin, 2002; Li and Verma, 2002).

With an objective to search for effective chemical agents for pancreatic cancer treatment, we have shown earlier that brucein D (BD) possesses potent antiproliferative and apoptogenic effects on human pancreatic cancer cells (Lau *et al*, 2008b). Moreover, we have recently reported that BD induces apoptosis in pancreatic cancer cells by activating the phosphorylation of p38-mitogen-activated protein kinase (MAPK) (Lau *et al*, 2009). Although the function of ROS in the activation of p38-MAPK (Yi *et al*, 2004; Lee *et al*, 2008; Chen *et al*, 2009), and the regulation of NF-*κ*B activity (Hayakawa *et al*, 2003; Fujioka *et al*, 2004), has been characterised in substances-induced apoptosis in various cancer cells, the involvement of ROS in BD-induced pancreatic cancer cell apoptosis is yet to be elucidated. Given that ROS have an important function in an array of biological responses and diverse signalling pathways, we hypothesised that BD activates pro-apoptotic pathways through the enhancement of ROS production and the inhibition of NF-*κ*B activity in pancreatic cancer cells. To test this hypothesis, we investigated (1) the function of oxidative stress and NF-*κ*B activity in BD-induced apoptosis in a pancreatic cancer cell line, PANC-1; (2) the involvement of ROS in regulating the p38-MAPK and NF-*κ*B signalling pathways in BD-treated PANC-1 cells; (3) the toxicity and anti-pancreatic cancer efficacy of BD in a pancreatic tumour xenograft animal model.

## Materials and methods

### Cell culture and BD treatment

Unless otherwise specified, the chemicals and reagents used in the project were obtained from Sigma-Aldrich (St Louis, MO, USA). PANC-1, a human pancreatic adenocarcinoma cell line obtained from American Type Culture Collection (Manassas, VA, USA), was grown in DMEM medium supplemented with 10% foetal bovine serum (Invitrogen, Grand Island, NY, USA), 4 mM L-glutamine, 4.5 g l^–1^ glucose, 1.5 g l^–1^ sodium bicarbonate, 100 U ml^–1^ penicillin and 10 U ml^–1^ streptomycin (Invitrogen) in a 5% CO_2_, 95% air humidified atmosphere. The procedure of the treatment of PANC-1 cells with BD was similar to that described in our earlier study (Lau *et al*, 2009).

**Figure 1:**
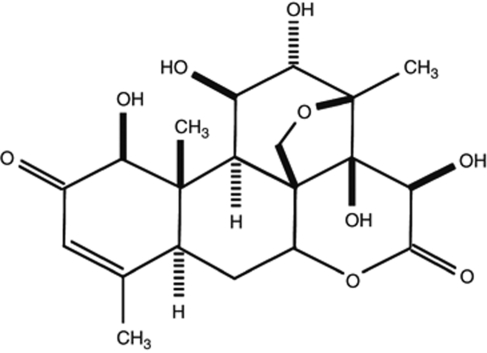
Chemical structure of BD, a C-20 quassinoid.

BD used in the present experiments was isolated from the fruit of *Brucea javanica* L. and its identity was confirmed by comparing its nuclear magnetic resonance and mass spectroscopy profiles with the published data (Yang *et al*, 1996), whereas its purity was calculated to be over 95% based on HPLC analysis (data not shown). Figure 1 shows the chemical structure of BD. For anti-oxidative experiments, an antioxidant, *N*-acetylcysteine (NAC; Sigma-Aldrich) was dissolved in phosphate-buffered saline (PBS) and incubated with the cells 1 h before BD treatment.

### Glutathione assay

Glutathione levels in PANC-1 cells were determined by an earlier described method (Ip *et al*, 2003; Chan and Leung, 2007). Briefly, PANC-1 cells were harvested and washed with PBS at the end of BD treatment. Trichloroacetic acid (25%) was mixed with PANC-1 cells at a ratio of 1 : 2 to precipitate protein. The mixture was subjected to centrifugation at 7000 *g* for 4 min at 4°C and the clear supernatant was obtained. The supernatant (200 *μ*l) was diluted with 0.1 M phosphate buffer (1 ml) and GSH assay was started by adding 3 mM 5-5′-dithiobis[2-nitrobenzoic acid] (50 *μ*l). The reaction was incubated at room temperature for 5 min and product measured by absorbance at 412 nm using a microplate spectrophotometer (Fluostar Optima, BMG Labtech, Durham, NC, USA). A standard curve was generated based on known concentration of GSH and was used to quantify the GSH content of each sample. Total GSH content was normalised by the protein concentration of PANC-1 cells.

### *In situ* detection of superoxide

Dihydroethidium (DHE) staining for superoxide was carried out as described earlier (Cheng *et al*, 2008). In brief, after BD treatment, the cells were immediately fixed with 4% paraformaldehyde (ICN, Irvine, CA, USA) for 30 min. Dihydroethidium (5 *μ*M) was dissolved in dimethyl sulphoxide and DAPI (1 : 1000, Jackson ImmunoResearch Laboratories, Burlingame, CA, USA) added to the slides and incubated at 37°C for 30 min. After washing with PBS, DHE staining was visualised using a confocal microscope (Leica DMLB, Wetzlar, Germany) equipped with a Leica DC 200 digital camera. Fluorescence intensity of DHE-stained PANC-1 cells was quantified using EZ-C1 Viewer 3.6 (Nikon, Tokyo, Japan).

### Western blot analysis

The whole-cell lysate of PANC-1 cells was obtained by lysing with CytoBluster protein extraction reagent (Novagen, Darmstadt, Germany), and stored at −20°C. The cytoplasmic and nuclear proteins were extracted using nuclear extraction kit (Imgenex Corporation, San Diego, CA, USA). In brief, the cells were washed with 5 ml ice-cold PBS-PMSF after BD treatment. Buffer was aspirated and cells collected using cell scraper with 5 ml ice-cold PBS-PSMF (Tsang *et al*, 2004). The cell pellets were obtained by centrifugation for 5 min at 1000 rpm at 4°C. Then the pellets were incubated with 1 ml hypotonic buffer on ice for 5 min, followed by addition of 50 *μ*l 10% detergent solution and vigorous vortexing for 10 s. After centrifugation at 14 000 rpm for 30 s at 4°C, the supernatant containing cytoplasmic protein and pellet containing the nuclear fraction were obtained. The nuclear pellet was resuspended in 100 *μ*l nuclear lysis buffer and vortexed vigorously. The suspension was then incubated at 4°C for 30 min on a rocking platform, followed by centrifugation at 14 000 rpm for 10 min at 4°C and the supernatant containing nuclear protein was obtained. The cytoplasmic and nuclear proteins were stored at −20°C and −80°C, respectively. Protein lysates were then resolved by SDS–PAGE for 1.5 h at 120 V and transferred electrophoretically to PVDF membrane for 1 h at 17 V. Membranes were blocked with 5% (w/v) non-fat dry milk in PBS-T (0.1% v/v Tween-20 in PBS) for 1 h and subsequently incubated overnight at 4°C with primary antibodies at the following concentrations: rabbit anti-p22^phox^ and p67^phox^ (Santa Cruz, Santa Cruz, CA, USA) [1 : 500], rabbit anti-phospho-p38-MAPK (Cell Signaling, Beverly, MA, USA) [1 : 1000], rabbit anti-p38-MAPK (Cell Signaling) [1 : 1000], rabbit anti-I*κ*B-*α* (Sigma-Aldrich) [1 : 500], rabbit anti-phospho-I*κ*B-*α* (Cell Signaling) [1 : 1000], rabbit anti-phospho-NF-*κ*B p65 (Cell Signaling) [1 : 1000] and mouse anti-*β*-actin (Sigma-Aldrich) [1 : 2500]. The membrane was incubated with horseradish peroxidase (HRP)-conjugated donkey anti-rabbit IgG or HRP-conjugated sheep anti-mouse IgG (Amersham Biosciences, Buckinghamshire, UK) for 1 h at room temperature. To verify equal loading of samples, the membranes were subsequently incubated with monoclonal antibody *β*-actin, followed by an HRP-conjugated sheep anti-mouse IgG. The protein bands were visualised by ECL western blotting detection reagents (Amersham Biosciences). For analysis of p38-MAPK, the cells were pre-incubated overnight in serum-free DMEM medium to remove any serum effects. The density of each band was determined by Leica IM computer software (Wetzlar, Germany).

### Electrophoretic mobility shift assay

Electrophoretic mobility shift assay (EMSA) was performed using a commercially available Gel Shift Assay System (Promega, Madison, WI, USA) based on our earlier study (Chan and Leung, 2007). Briefly, the nuclear extract (5 *μ*g) or HeLa cell extract (positive control) was incubated with ^32^P-labelled oligonucleotide probe (5′-AGT TGA GGG GAC TTT CCC AGG C-3′) in the presence of binding buffer [4% glycerol, 1 mM MgCl_2_, 0.5 mM EDTA, 0.5 mM dithiothreitol, 50 mM NaCl, 10 mM Tris–HCl, pH 7.5 and 50 *μ*g ml^–1^ poly(dI-dC)·poly(dI-dC)] at room temperature for 20 min. After incubation, the DNA–protein complex was resolved by 4% non-denaturing polyacrylamide gel at 220 V. The gel was dried and exposed to X-ray film overnight at −80°C and the band densities were quantified by phosphorimager analysis using FlouroChem8000 (Alpha Innotech, San Leandro, CA, USA).

### RNA extraction, reverse transcription and real-time PCR

Gene expression was detected by RT–PCR. The QIAGEN RNeasy Mini Kit (QIAGEN, Valencia, CA, USA) was used to extract total RNA from PANC-1 cells from different treatment groups as described earlier (Leung *et al*, 1999; Lam and Leung, 2002). First-strand cDNA was generated from 2 *μ*g of total RNA using oligo(dT) primer. *β*-actin cDNA was amplified as an endogenous control. Real time–PCR was performed using iQ5 Multicolor RT–PCR Detection System (Bio-Rad, Hercules, CA, USA) and the following steps: 95°C for 2 min; 95°C for 30 s, 63°C for 45 s, 95°C for 1 min (40 cycles), and 72°C for 10 min. The results are normalised to *β*-actin and expressed as relative mRNA expression level according to the 2-CT method (Livak and Schmittgen, 2001). Melting curve analysis was performed to confirm amplification specificity of the PCR products. Primer sequences for amplification of the human genes were as follows: bcl-2 forward, 5′-CGA GTT CAG TGG AGG AGA CC -3′ bcl-2 reverse, 5′- GCC TAA GGA AGG CAG CTA GG -3′ XIAP forward, 5′- CCT TGG GAA CAA CAT GCT AAA -3′ XIAP reverse, 5′- ACC AGA CAC TCC TCA AGT GAA -3′ *β*-actin forward, 5′-TGT CCA CCT TCC AGC AGA TGT-3′ and *β*-actin reverse, 5′-CGG ACT CGT CAT ACT CCT GCT T-3′ (Invitrogen, Hong Kong, China).

### *In vivo* evaluation of tumour inhibition

Six-week-old male BALB/c nude mice were supplied by the Laboratory Animal Services Centre of The Chinese University of Hong Kong. The animals were housed under pathogen-free conditions in specifically designed air-controlled rooms with a 12-h light/dark cycle and fed with food and sterile water *ad libitum*. The care and use of the animals were in compliance with the institutional guidelines, and the experimental procedures were approved by the Animal Experimentation Ethics Committee of the CUHK (Ref. 08/032/MIS).

Xenografts were formed by subcutaneous inoculation of 5 × 10^6^ CAPAN-2 cells (kindly provided by Dr Chaoyang Chen of the Faculty of Medicine, CUHK) in PBS. Treatment was initiated once the tumours reached a mean diameter of 8–10 mm. Mice were then randomised into different treatment groups of four mice each. Brucein D dissolved in 1.68% *γ*-cylodextrin was administered at various concentrations (0.375, 0.75 and 1.5 mg kg^–1^) once daily by tail vein injection for 10 consecutive days. The selection of the drug concentrations was based on the results of an earlier pilot study using a range of concentrations, that is 12, 6, 3, 1.5, 0.75 and 0.375 mg kg^–1^ per day, which showed that the concentrations of 1.5, 0.75 and 0.375 mg kg^–1^ per day elicited significant beneficial effects on the animal without causing any toxicity. Tumour volume (TV) and body weight were monitored daily and the TV was calculated by using the formula TV=4/3*πr*^3^, where *r* is half of the mean tumour diameter, measured in at least two directions (Sloss *et al*, 2008).

### Plasma-specific enzyme level measurement

At the end of the treatment, the blood of the mice was obtained by cardiac puncture. After centrifugation, plasma was collected and stored at −20°C. Plasma aspartate transaminase (AST) and plasma alanin transminase (ALT) for liver damage, and plasma lactate dehydrogenase (LDH) and plasma creatine kinase (CK) for heart damage were analysed according to the manufacturer's instructions (Biosystems S.A., Barcelona, Spain).

### Histological examination

At the end of the treatment, the mice were euthanised by cervical dislocation. Heart, liver and kidney were withdrawn and fixed in ice-cold 4% PFA in PBS at 4°C overnight. The fixed tissues were processed by Shando Pathcentre Tissue Processor (Thermo Scientific, Waltham, MA, USA). After dehydration, the tissues were embedded in molten paraffin (Merck, Germany) at 60°C and cast at room temperature. The paraffin-embedded tissue blocks were sectioned at 5 *μ*m using a biocut rotary microtome machine (Reichert-Jung, Germany) onto gelatin-coated slides. The sections were then de-waxed by xylene and subsequently rehydrated by ethanol gradient (absolute to 70% ethanol, each for 30 s). After several washings, the sections were stained with haematoxylin for 5–8 min to locate the nucleus. Colour of haematoxylin was adjusted by rinsing with 1% acid alcohol and Scott's tap water. Then the sections were stained with 1% eosin for 5 min to visualise the cytoplasm. The slides were subjected to dehydration by ethanol gradient and xylene (three times, each for 30 s), and mounted using microscopic rapid-mounting media (Merck, Germany). Histological changes were examined under a light microscope (DFC 490, Olympus, Japan).

### Statistical analysis

Multiple comparisons between data sets were performed using one-way analysis of variance followed by Dunnett's *post hoc* test. Statistical analyses were conducted using a GraphPad Prism 3.02 software package (GraphPad Software Inc., San Diego, CA, USA).

## Results

### Effects of BD on cellular glutathione concentration and superoxide production

**Figure 2:**
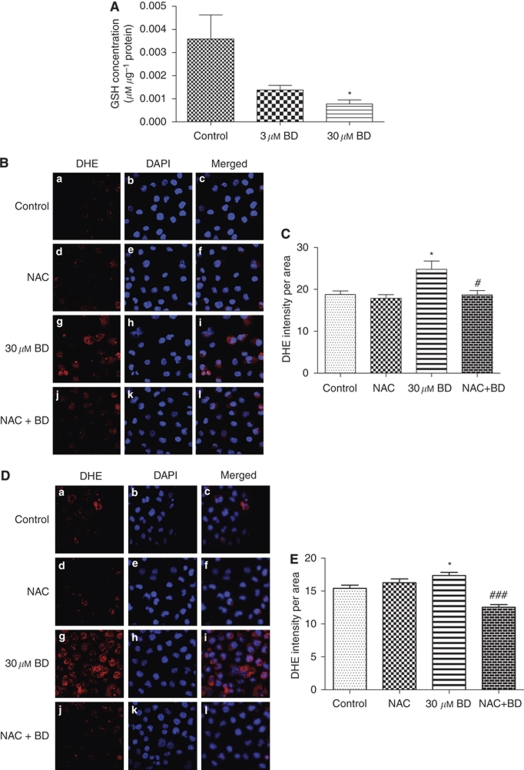
Effects of BD on redox balance in PANC-1 cells. (**A**) BD attenuates antioxidant levels in PANC-1 cells. Cells were treated with BD for 2 h and GSH levels were determined. (**B**, **D**) Representative diagram showing dihydroethidium (DHE) staining of PANC-1 cells after treatment with vehicle (a–c), 250 *μ*M NAC (d–f), 30 *μ*M BD (g–i) and 250 *μ*M NAC + 30 *μ*M BD (j–l) for 1 or 2 h. Cells were fixed in 4% PFA and then incubated with DHE to detect the superoxide production (red). Nuclei were counterstained with DAPI (blue). Merged images are shown for each treatment (c, f, i and l). (**C**, **E**) Effects of BD on superoxide production in PANC-1 cells. Cells were pre-incubated with 250 *μ*M NAC for 1 h and then treated with 30 *μ*M BD for 1 or 2 h. The intensity of DHE staining was measured. ^*^P<0.05 *vs* vehicle-control group; ^#^*P*<0.05 and ^###^*P*<0.001 *vs* 30 *μ*M BD-treatment group. The results were obtained from at least three independent experiments. (The colour reproduction of this figure is available on the html full text version of the manuscript.)

After treatment with 3 and 30 *μ*M BD for 2 h, the GSH levels in PANC-1 cells decreased significantly (Figure 2A). The depletion of GSH levels in BD-treated PANC-1 cells showed a dose-dependent trend, and the GSH concentrations in both 3 and 30 *μ*M BD-treated cells were significantly lower than that of the control cells. Next, the production of superoxide in BD-treated PANC-1 cells was measured by *in situ* DHE staining. PANC-1 cells treated with 30 *μ*M BD for 1 and 2 h showed enhanced DHE staining in the nucleus of the cells (Figure 2B and D), indicating increased production of superoxide anions. Quantification of the fluorescence intensity revealed that 30 *μ*M BD significantly accentuated the production of superoxide in both treatment time periods in PANC-1 cells (Figure 2C and E). Elevation of superoxide-free radical generation induced by BD was significantly inhibited by pre-incubation of the cells with an antioxidant, NAC (250 *μ*M). Our results indicate that BD is able to induce ROS by depleting GSH and enhancing superoxide generation in PANC-1 cells.

### Role of NADPH oxidase in BD-treated PANC-1 cells

**Figure 3:**
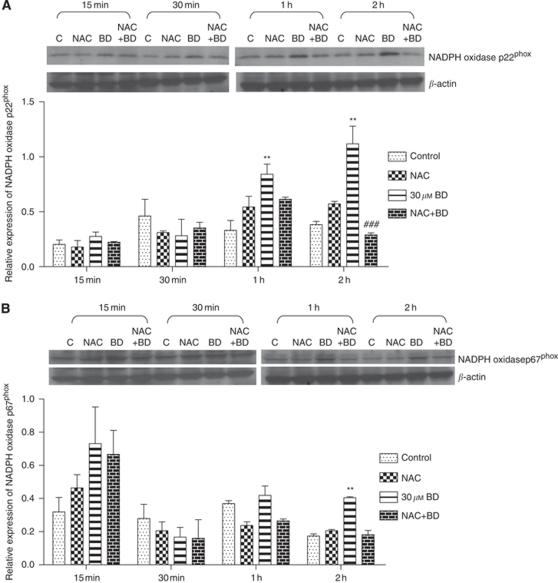
Brucein D-treated results in an increase in the expression of NADPH oxidase. Effects of BD on the protein expression of NADPH oxidase p22^phox^ (**A**) and p67^phox^ (**B**) in PANC-1 cells in the absence and presence of 250 *μ*M NAC. Representative images and statistical analysis of band intensity are shown in upper and lower panels, respectively. The results were obtained from at least three independent experiments. ^**^P<0.01 *vs* vehicle-control group; ^###^*P*<0.001 *vs* 30 *μ*M BD-treatment group. The results were obtained from at least three independent experiments.

NADPH oxidase activity was measured in BD-treated PANC-1 cells for different incubation periods (15 min to 2 h). Western blot results indicated that treatment with 30 *μ*M BD increased protein expression of both p22 and p67 subunits of NADPH oxidase in PANC-1 cells (Figure 3A and B, respectively). When compared with the control, the expression of p22^phox^ NADPH oxidase increased significantly after treatment with 30 *μ*M BD for 1 and 2 h. However, a significant increase of p67^phox^ NADPH oxidase seemed only after 2 h incubation with BD. Pre-treatment of PANC-1 cells with 250 *μ*M NAC effectively prevented BD-mediated augmentation in the expression of p22^phox^ and p67^phox^ NADPH oxidase.

### Effects of BD-induced ROS on p38-MAPK activation

**Figure 4:**
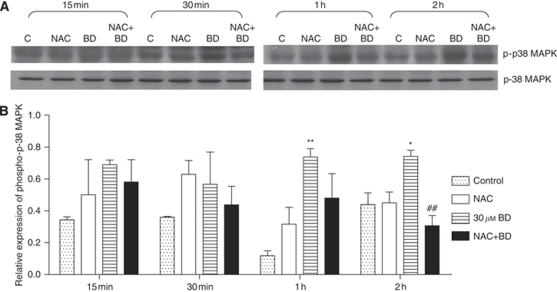
Effects of pre-treatment with NAC of the phosphorylation of p38-MAPK after treatment with BD. Cells were pre-treated with 250 *μ*M NAC for 1 h, then incubated with 30 *μ*M BD for 15 min to 2 h. (**A**) Western bolt analysis of samples using total p38 (p38-MAPK) and phosphorylation-specific p38 (p-p38-MAPK) antibodies. The results were obtained from three independent experiments. (**B**) Quantitative analysis of western blot band intensities. ^*^P<0.05 *vs* vehicle control, ^**^P<0.01 *vs* control and ^##^*P*<0.01 *vs* 30 *μ*M BD treatment.

To examine the function of BD-induced ROS in mediating the activation of p38-MAPK in PANC-1 cells, the expression of p38-MAPK protein in each of the treatment groups was analysed. A distinct phosphorylation of p38-MAPK in PANC-1 cells was observed after 30 *μ*M BD treatment for 1 and 2 h (Figure 4). Pre-treatment with 250 *μ*M NAC effectively abolished the phosphorylation of p38-MAPK in BD-treated cells, indicating the requirement of ROS for activation of p38-MAPK.

### Involvement of the NF-*κ*B signalling pathway in BD-treated PANC-1 cells

**Figure 5:**
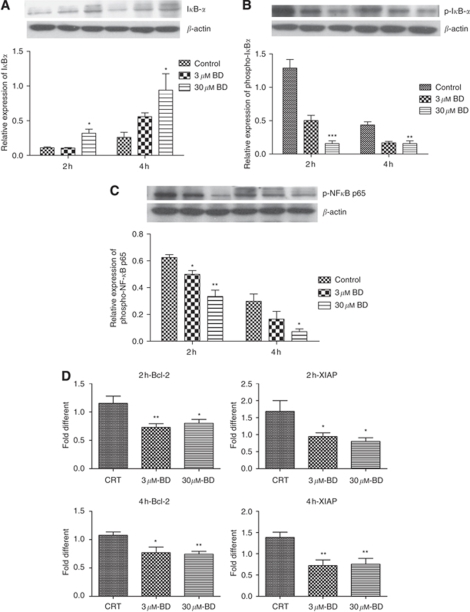
Effects of BD on the activation of NF-*κ*B. PANC-1 cells were treated with 3 and 30 *μ*M BD for 2–4 h. (**A**, **B**) Western blots of cytoplasmic protein extracts (20 *μ*g per lane) using antibodies specific for I*κ*B-*α* and phospho-I*κ*B-*α*. (**C**) Western blots of nuclear protein extracts (20 *μ*g per lane) using a phospho-NF-*κ*B p65 antibody. (**D**) RT–PCR analysis for the mRNA expression of NF-*κ*B-regulated genes, bcl-2 and XIAP. ^*^P<0.05 and ^**^P<0.01 *vs* vehicle control. The results were obtained from three independent experiments.

To determine the effects of BD on the NF-*κ*B pathway, PANC-1 cells were treated with BD for 2 or 4 h. The levels of phosphorylated I*κ*B-*α* in PANC-1 cells significantly decreased with increasing concentrations of BD (Figure 5A). Treatment with 30 *μ*M BD significantly accentuated I*κ*B-*α* protein levels (Figure 5B), suggesting that BD suppressed the growth of PANC-1 cells by inhibiting NF-*κ*B activation through augmenting I*κ*B protein levels. In addition, phospho-NF-*κ*B p65 protein expression was examined in PANC-1 cell nuclear fractions. The result showed that phospho-NF-*κ*B p65 expression was significantly attenuated in the nucleus after treatment with 3 and 30 *μ*M BD for 2 or 4 h (Figure 5C).

Nuclear factor-*κ*B-regulated genes were also examined to determine whether BD affected their expression. The mRNA expression of anti-apoptotic and NF-*κ*B target genes, bcl-2 and XIAP, was significantly decreased after BD treatment (Figure 5D). These results indicate that inhibition of NF-*κ*B activity by BD-treatment affects the transcription of anti-apoptotic genes, resulting in decreased resistance to apoptosis in PANC-1 cells.

### Effects of BD-induced ROS in NF-*κ*B signalling activity

**Figure 6:**
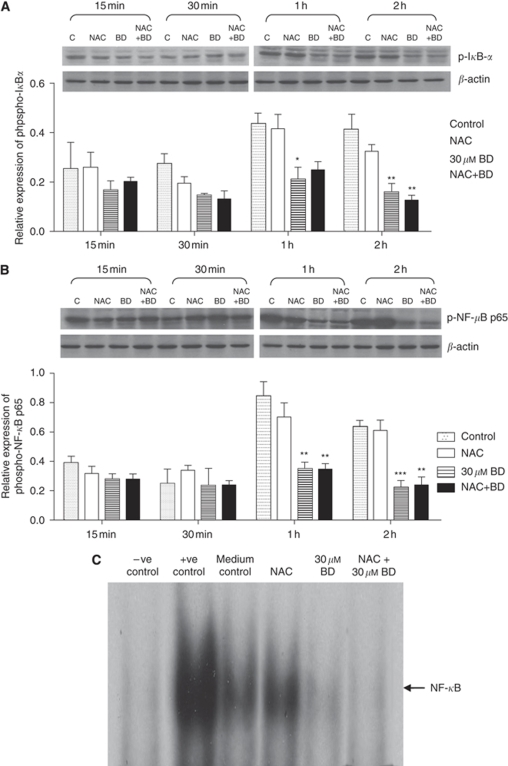
Brucein D-induced inhibition of NF-*κ*B activation is independent of ROS in PANC-1 cells. Cells were pre-treated with 250 *μ*M NAC or vehicle for 1 h, then incubated with 30 *μ*M BD for 15 min to 2 h. Western blot analysis was performed on cytoplasmic lysate using anti-phospho-I*κ*B*α* antibodies (**A**) and nuclear lysate using anti-phospho-NF-*κ*B p65 antibodies (**B**). ^*^P<0.05, ^**^P<0.01 and ^***^P<0.001 *vs* vehicle control. The results were obtained from three independent experiments. Quantitative analysis of band intensities is shown in lower panels, respectively. (**C**) Representative image showing the results from EMSA of nuclear extracts of PANC-1 cells after BD treatment for 2 h. Lane 1: distilled water (negative control); Lane 2: HeLa cell extract (positive control); Lane 3: vehicle control; Lane 4: 250 *μ*M NAC treatment; Lane 5: 30 *μ*M BD treatment; Lane 6: 250 *μ*M NAC + 30 *μ*M BD treatment. Similar results were obtained in three independent experiments.

The phosphorylation of I*κ*B-*α* significantly decreased after BD treatment for 1 or 2 h (Figure 6A). Similarly, NF-*κ*B p65 activity was significantly reduced in the nuclear portion of PANC-1 cells when treated with BD for 1 or 2 h. To evaluate the involvement of BD-induced ROS in NF-*κ*B activation, PANC-1 cells were pre-treated with NAC. Interestingly, no difference was observed in the levels of phospho-I*κ*B-*α* and phospho-NF-*κ*B p65 between the BD alone treated cells and that pre-treated with NAC (Figure 6A and B). Cells pre-treated with NAC alone (without BD treatment) exhibited no change in the activity of both phosphor-I*κ*B-*α* and phosphor-NF-*κ*B p65 compared with the vehicle-control group.

The ability of BD to inhibit the DNA-binding activity of NF-*κ*B in PANC-1 cells was also examined by EMSA. As shown in Figure 6C, constitutive NF-*κ*B binding was evident in exponentially growing PANC-1 cells when cultured in medium only. Treatment with 30 *μ*M BD for 2 h markedly inhibited NF-*κ*B DNA-binding activity in PANC-1 cells. Electrophoretic mobility shift assay results confirmed that pre-treatment of cells with NAC did not affect the DNA-binding activity in cells treated with either vehicle or BD.

### BD inhibits the growth of CAPAN-2 human pancreatic tumour xenografts *in vivo*

**Figure 7:**
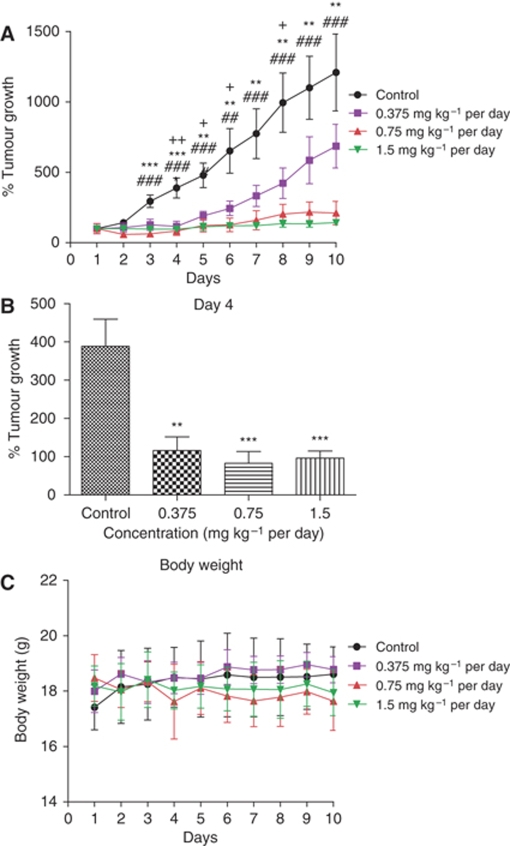
*In vivo* analysis of the anti-tumour effects of BD. CAPAN-2 cells were xenografted by subcutaneous inoculation into nude mice. (**A**) Mice were treated with BD daily at the indicated concentrations through i.v. administration for 10 consecutive days. The tumour volume (TV) of each mouse was determined using the formula: TV=4/3*πr*^3^; *n*=4, where ^+^*P*<0.05 and ^++^*P*<0.01 comparing 0.375 mg kg^–1^ per day *vs* vehicle control; ^**^P<0.01 and ^***^P<0.001 comparing 0.75 mg kg^–1^ per day *vs* vehicle control; ^##^*P*<0.01 and ^###^*P*<0.001 comparing 1.5 mg kg^–1^ per day *vs* vehicle control. (**B**) Effects of BD on tumour xenograft growth in nude mice after 4 days of treatment at the indicated concentrations. The percentage of tumour growth was calculated after 4 days BD treatment; *n*=4, ^**^P<0.01 and ^***^P<0.001 *vs* vehicle control. (**C**) The body weight of each mouse was measured for 10 days.

Pancreatic tumours were successfully established in nude mice after transplanting human pancreatic adenocarcinoma CAPAN-2 cells. In Figure 7A, the size of the xenograft tumours formed in the control-treatment mice were markedly larger than that of the 1.5 mg kg^–1^ per day BD-treated mice. Daily TV measurements revealed that the tumour sizes in BD-treated mice were, in general, smaller than those of the vehicle-control group. These data indicate that BD is able to significantly suppress the tumour growth in a xenograft model in a dose-dependent manner. It should be noted that the tumours grew exponentially in the vehicle-control mice. When exposed to BD at the concentrations of 0.75 and 1.5 mg kg^–1^ body weight, the rate of tumour growth in the xenograft mice remained at a very low level throughout the entire treatment period. It is worth noting that mice treated with BD started to show significant inhibition of tumour growth on day 4 (Figure 7B).

### *In vivo* toxicity test of BD

Mice treated with BD intravenously at dose as high as 1.5 mg kg^–1^ for 10 days did not show any drug-related side effects. The body weight of mice with BD injection for 10 consecutive days did not show significant differences to that of the *γ*-cylodextrin-control group (Figure 7C). Plasma was collected at the end of the experiment and the presence of heart and liver-specific enzymes in plasma because of organ damage was assessed. No significant difference was found between *γ*-cylodextrin and BD-treatment groups regarding the activities of plasma enzymes CK, LDH, ALT and AST (Supplementary Figure 1). Furthermore, histological examination revealed no apparent alterations in the structural organisation of the heart, liver and kidney tissues between the BD-treated and control groups (Supplementary Figure 2), suggesting that BD treatment at 1.5 mg kg^–1^ for 10 days caused no toxicity to the heart, liver and kidney tissues in the tumour-bearing mice.

## Discussion

Our earlier studies identified BD, a quassinoid found abundantly in Chinese medicine Fructus Bruceae javanica, to exert potent antiproliferative activity in cultured human pancreatic adenocarcinoma cells through the induction of apoptosis involving activation of p38-MAPK signalling (Lau *et al*, 2008b, 2009). In this study, we attempted to further elucidate the underlying mechanism by which BD mediates anti-proliferative action on pancreatic cancer cells. Our data have shown that BD mediates activation ROS-regulated p38-MAPK phosphorylation, whereas inhibiting NF-*κ*B anti-apoptotic activity in pancreatic cancer cells.

The intracellular redox status reflects a precise balance between oxidative stress and the endogenous thiol-buffering system. It has been shown that an imbalance of the intracellular redox state towards oxidative stress triggers downstream cellular events, such as alteration of mitochondrial function and cell signalling pathways leading to apoptotic cell death (Zhang *et al*, 2004). Our results showed that in pancreatic cancer cells, treatment with BD caused reduction of intracellular GSH in a does-dependent manner. Glutathione is the most abundant bulk antioxidant, which prevents apoptosis and maintains viability of the cells (Slater *et al*, 1995). PANC-1 cells deprived of GSH on exposure to BD became more vulnerable to oxidative stress when their ability to scavenge and detoxify the various metabolically generated reactive oxygen intermediates was impaired. In addition, BD treatment of PANC-1 cells resulted in an increased generation of superoxide-free radicals. Pre-treatment of PANC-1 cells with NAC, an antioxidant capable of inhibiting apoptosis induced by ROS (Yang *et al*, 2007), diminished the production of superoxide induced by BD. We further showed that membrane NADPH oxidase activity was present in PANC-1 cells, and was elevated with BD treatment. Non-mitochrondrial NADPH oxidase is a multi-component complex consisting of two membrane proteins, gp91^phox^ and p22^phox^, and four cytosolic factors, p47^phox^, p67^phox^, p40^phox^ and Rac (Babior *et al*, 2002). The gp91^phox^ and p22^phox^ proteins comprise the catalytic centre of the enzyme flavocytochrome b_558_. Activation of the enzyme catalyses the conversion of molecular oxygen (O_2_) to superoxide-free radical (O_2_^•−^), which generates oxidative stress (Griendling *et al*, 2000). Our data showed that PANC-1 cells expressed the key components of NADPH oxidase p22^phox^ and p67^phox^, and after treatment with BD, protein expression of both p22^phox^ and p67^phox^ were up-regulated. The up-regulation of p22^phox^ and p67^phox^ by BD was effectively abolished by pre-treatment with NAC. These data indicate that NADPH oxidase is a source of ROS on BD treatment.

Considerable evidence suggests that ROS have an important function in key intracellular signal transduction pathways for a variety of cellular process (Aslan and Ozben, 2003; Poli *et al*, 2004; Chiarugi, 2005). Recently, ROS has been proposed to be involved in tumour metastasis (Radisky *et al*, 2005; Storz, 2005). One of the important downstream signals involved in tumour invasion is activation of MAPKs, including ERK, p38 and JNK (Berra *et al*, 1995; Schonwasser *et al*, 1998). All MAPKs are activated by dual phosphorylation of threonine and tyrosine motifs within the activation loop. Once activated, MAPKs translocate to the nucleus in which they phosphorylate target transcription factors. Our earlier work unequivocally showed that treatment with BD increased phosphorylation of p38-MAPK, and subsequently activated the caspase cascade in PANC-1 cells leading to cellular apoptosis (Lau *et al*, 2009). Given that BD is able to induce oxidative stress and activate NADPH oxidase, the effect of NAC on p38-MAPK activation in PANC-1 cells was assessed. Immunoblotting analyses showed that pre-treatment of NAC markedly abrogated p38-MAPK activation in BD-treated cells, suggesting that ROS was upstream of p38-MAPK activation.

Nuclear factor-*κ*B has been reported to be activated in various tumours, such as human leukaemia and lymphoma, the lung, breast, colon and pancreatic carcinomas (Rayet and Gelinas, 1999; Karin and Lin, 2002; Wu and Kral, 2005; Braeuer *et al*, 2006). The p65 subunit of NF-*κ*B is constitutively activated in most human pancreatic cancer tissues and cell lines (Wang *et al*, 1999), and this activation has been shown to have important functions in tumourigenesis and liver metastasis of pancreatic adenocarcinoma (Fujioka *et al*, 2003). Therefore, inhibition of NF-*κ*B has become a major strategy in anti-cancer therapy for pancreatic tumours.

According to our data, BD treatment inhibited phosphorylation and degradation of the inhibitory subunit, I*κ*B-*α*, thereby sequestering NF-*κ*B and preventing nuclear translocation and DNA binding. The important function of NF-*κ*B in protecting cells against diverse apoptotic stimuli including chemo- and radiotherapy is well known. Anti-apoptotic NF-*κ*B-regulated genes include genes that encode bcl-2-like proteins and IAP proteins (IAP-1, IAP-2, XIAP) (Barkett and Gilmore, 1999). Increased activation of p65 in PANC-1 cells was abolished by BD treatment, leading to a decrease in the transcription of bcl-2 and XIAP.

It has been proposed that ROS are involved in the activation of NF-*κ*B. Certain types of compounds with antioxidant properties block NF-*κ*B activation and most agents that activate NF-*κ*B are known to trigger ROS formation. For instance, NF-*κ*B activation can be induced by treatment with H_2_O_2_ in certain cell lines. However, the contribution of redox regulation in NF-*κ*B activation is a topic for intense debate (Brennan and O'Neill, 1995; Korn *et al*, 2001). In addition, several research groups have reported that H_2_O_2_-induced NF-*κ*B activation is highly cell type dependent (Anderson *et al*, 1994; Bowie *et al*, 1997; Li and Karin, 1999). Thus, we initially hypothesised that ROS induced by BD treatment serve as secondary messengers mediating NF-*κ*B activity. Surprisingly, pre-treatment of the most widely used antioxidant, NAC, did not show any regulatory effect on NF-*κ*B in PANC-1 cells with or without BD treatment. Hayakawa *et al* (2003) showed that endogenous ROS produced through Rac/NADPH oxidase do not mediate NF-*κ*B signalling. It is thus plausible that the inhibition of NF-*κ*B activity was independent of the ROS induced by BD treatment.

In drug development, it is essential to evaluate the toxicity and potency of drug candidates *in vivo*. In our current project, we evaluated the anti-tumour efficacy of BD in a nude mice model xenografted with CAPAN-2 human pancreatic cancer cells. We found that BD was able to inhibit the tumour growth without causing mortality, significant weight loss or other noticeable side effects on the heart, liver and kidney tissues (Supplementary Figures 1 and 2). These observations are in agreement with our *in vitro* studies showing that treatment of pancreatic cancer cells with BD resulted in a concentration-dependent induction of apoptosis. The potent anti-tumour activity of BD together with the absence of toxicity renders this plant-derived quassinoid a promising drug candidate in pancreatic cancer chemotherapy. It seems that further in-depth pre-clinical animal studies and, ultimately, clinical trials on BD is warranted for development of this chemical into anti-cancer pharmaceutical.

In conclusion, this study is the first report to delineate the mechanistic pathways associated with BD-mediated apoptosis in pancreatic cancer cells. Brucein D depleted the intracellular GSH levels, favouring the onset of apoptosis by passively allowing oxidative stress to build up. Oxidative stress generated by NADPH oxidase activation leads to p38-MAPK activation, causing apoptosis in pancreatic cancer cells. In addition, BD treatment inhibits anti-apoptotic gene expression by blocking NF-*κ*B activation, which may increase cell susceptibility to cytotoxic agents. Taking together the *in vitro* and the preliminary, but promising, *in vivo* data, we believe that BD has good potential for further development into a clinical treatment for pancreatic cancer.
