# Supplementary material for: Role of reactive oxygen species in brucein D-mediated p38-mitogen-activated protein kinase and nuclear factor-κB signalling pathways in human pancreatic adenocarcinoma cells
Source: Br J Cancer. 2010 Jan 12;102(3):583–93. doi: 10.1038/sj.bjc.6605487 (PMC2822930; doi:10.1038/sj.bjc.6605487)
Supplement: Supplementary Figures 1 and 2 [file 6605487x1.doc]

**SUPPLEMENTARY FIGURE 1**

Figure S1. Effects of BD on the plasma enzyme activity of CK, LDH, AST and ALT in CAPAN-2 bearing nude mice after 10 days treatment. The mice were sacrificed after treatment and blood was withdrawn from the heart. Enzyme concentrations (U/L) in the sample was calculated using an equation U/L = ΔA/min x Vt x 10^6^/ε x Vs, where ε = molar absorbance of NADPH; Vt = total reaction time; Vs = sample volume.

**SUPPLEMENTARY FIGURE 2**

Figure S2. Histology of heart, liver and kidney tissue of the control and BD-treated mice. Nude mice with CAPAN-2 cell xenografts were treated with or without BD (1.5 mg/kg/day) for 10 days, and histological section was performed on heart, liver and kidney tissue at the end of experiment.
